# Vision guardians for community eye health: the LV Prasad Eye Institute model in India

**Published:** 2022-09-20

**Authors:** Srinivas Marmamula, Rajashekar Varada, Jachin D Williams, Rohit C Khanna

**Affiliations:** Associate Director, Public Health Research and Training: GPR ICARE, LV Prasad Eye Institute, Hyderabad, India.; Network Associate Director: GPR ICARE, LV Prasad Eye Institute, Hyderabad, India.; Associate Public Health Specialist: GPR ICARE, LV Prasad Eye Institute, Hyderabad, India.; Network Director: GPR ICARE, LV Prasad Eye Institute, Hyderabad, India.

**Vision guardians recruited from the local community can engage with people to support their eye care in the long term.**

Vision loss affects over a billion people worldwide.^1^ Although over 90% of vision loss is avoidable, several barriers limit the availability and uptake of services.^2^ The World Health Organization has adopted ‘universal eye health’ as a part of universal health coverage, which aims to ensure that “all people have access to *promotive, preventive, curative and rehabilitative health services*, of sufficient quality to be effective, while also ensuring that people do not suffer financial hardship when paying for these services.”^3^

**Figure:**
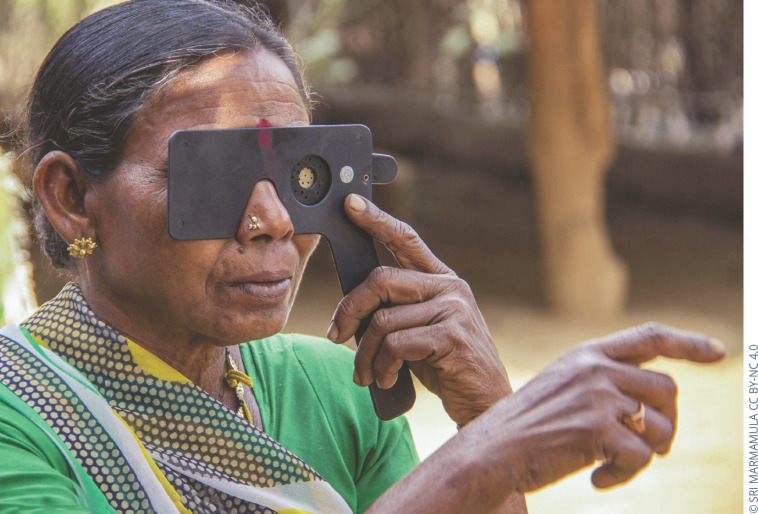
A patient responding to a vision chart. india

Community engagement is vital for the successful implementation of health interventions at a place where people need services. With this in mind, LV Prasad Eye Institute (LVPEI) developed a pyramidal eye care model which has vision guardians as its foundation (Figure 1).

Vision guardians are the part of the eye care system that is closest to the community. Vision guardians form an essential link that connects eye care services with the community, thereby providing a framework for community engagement on a continuous basis.^4^,^5^

Vision guardians are recruited from the local community. They are people who know the community and are known in the community; this promotes mutual trust and makes their work much easier. Each vision guardian looks after the eye health of about 5,000 community members. Their activities include:

- door-to-door vision screening to identify people with eye health issues
- eye health screening in schools
- referring people to the next level of care
- checking that patients with sight-threatening eye conditions have attended their referral appointments
- monitoring patients after surgery
- supporting community health activities, including health promotion and awareness programmes
- checking up on vulnerable individuals
- engaging with all stakeholders in the community: government and non-government bodies as well as village groups, including village health committees.

**Figure 1:**
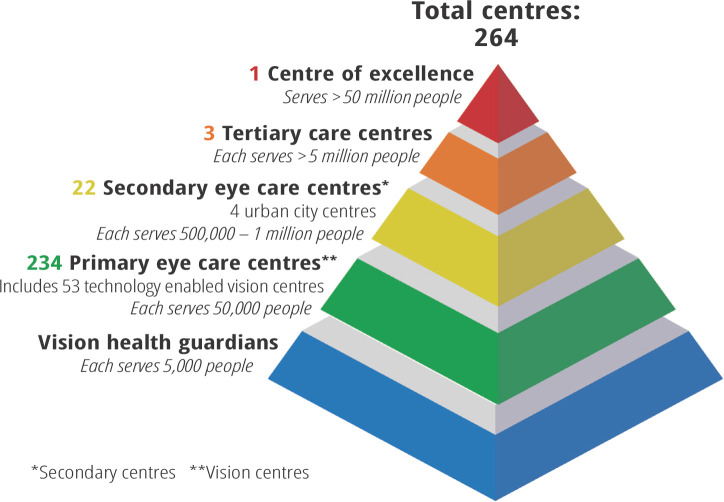
LV Prasad Eye Institute eye care service delivery pyramid

## Selection, recruitment, and training of vision guardians

Vision guardians are either volunteers who work part time, or are employed full time (and paid) by LV Prasad Eye Institute (LVPEI). Community organisations identify potential candidates from the local area for enrolment as vision guardians. Community involvement in the selection and recruitment of vision guardians promotes local ownership and acceptance of vision guardians by the community, all of which is essential for the success of this initiative.

Typically, women aged 18 and older who can write in the local language, are willing to travel in the field, and have good communication skills, are preferred. However, nobody is excluded on the basis of their gender, and there are instances where men who have met the prerequisites have been recruited.

Vision guardians are trained in:

- understanding the structure and functions of the eye
- identifying common conditions that cause vision loss in adults and children
- vision screening, basic torchlight examination, and documentation^6^
- basic record keeping (for tracking referrals)
- counselling.

The training is predominantly hands-on and conducted over one week. A refresher course is provided once a year.

## Monitoring indicators for vision guardians

Monitoring often improves outcomes and also ensures that the programme is moving in the right direction. Monitoring indicators are used to track performance, identify challenges, and provide additional training as and when required. For example, if the referral rate (%) is too low or too high for a particular vision guardian as compared to others in the region, it may be due to some issue with the screening protocol or due to selective screening of individuals, and may warrant further investigation and an appropriate resolution. A full-time supervisor is appointed for every ten vision guardians and is responsible for their routine monitoring and reporting from the field.

The parameters for monitoring vision guardians include the following:

- **Population coverage (%)**. Proportion of people screened at the household level in the catchment population
- **Referral rate (%)**. Proportion of people identified with eye problems and referred to the next level of care from among those screened
- **Referral uptake (%)**. Proportion of people seeking the next level of care from among those referred
- **Eye health promotion**. Number of awareness sessions conducted per month
- **Community engagement**. Number of community visits and interactions with stakeholders per month, including participation in local committee meetings in the community.

There are no specific monitoring indicators for volunteer vision guardians, except for regular communication through updates on referral of patients from their area and post-operative surveillance. Offering volunteers (and their families) incentives such as free eye care services, and involving them in events to celebrate the anniversary of the eye centre and World Sight Day, has been found to motivate them to continue their contribution as vision guardians. Winning societal recognition is another motivating factor for volunteering as vision guardians.

In addition, vision guardians can help to identify other disabilities alongside assessing eye health in vulnerable populations.^7^ For instance, in Rayagada, a tribal district of Odisha, over 100,000 people were screened at their doorsteps and referred to the next level of care. In addition to 9.4% of the people screened who had vision impairment, 2.8% of those screened were found to have other disabilities and were referred.^7^

**LVPEI Experience: Community Linked Initiative Project (CLIP)**

LVPEI implemented the Community Linked Initiative Project in one of its catchment areas to demonstrate the application of the vision guardian concept in eye health.

**Project summary**

**Location:** Jainad sub-district in Adilabad district in Telangana (52 villages)

**Total population:** 47,904 (Census 2011)

**Population covered:** 38,829 (81.1% coverage)

**Number of vision guardians:** 23 (all women volunteers)

**Duration:** 3 years (2011–2014)

**Key statistics**

- 38,829 people screened; 3,616 (9.3%) people identified with eye problems and referred
- 8,646 children screened through school screening programmes; 622 (7.2%) children identified with eye problems and referred
- 2,171 pre-primary school children (at Anganwadi centres) screened; 55 (2.5%) children identified with eye problems and referred
- spectacles dispensed to 1,472 people
- sight restoration surgeries performed on 472 people
- 30 people with irreversible visual impairment rehabilitated
- over 100 awareness programmes conducted.

At the end of the three-year project, the vision guardians have continued to support eye health initiatives through referrals and follow-up care. They have continued as volunteers and are the link with the local communities.

## Key challenges and way forward

A strong service delivery base is essential for the successful implementation of the vision guardian programme. Referral linkages, the capacity to recruit and train vision guardians, and a robust system for regular monitoring are important elements for the success of the programme. Vision guardians should be considered as an extended arm of service delivery. Over time, vision guardians, irrespective of whether they are paid or are volunteers, will become the ‘go-to’ persons in the catchment community for eye health issues.

**Figure:**
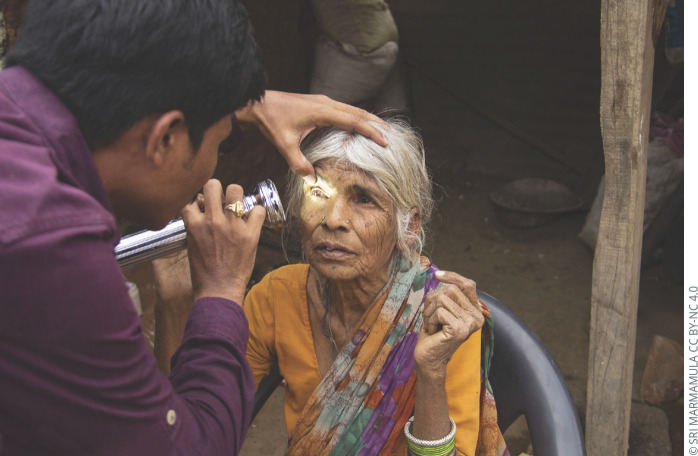
An eye examination using a torch. india

In our experience, using the services of volunteer vision guardians is less expensive, but inconsistent in terms of the output delivered as volunteers’ commitment tends to wane over time. A paid vision guardian system is more expensive in terms of programme costs, but more consistent in delivering output for lasting impact. Currently, LVPEI has over 70 paid vision guardians in various community activities. The number of volunteer vision guardians at any given time is not fixed. LVPEI is considering a study to assess the impact of paid vision guardians and volunteer vision guardians in order to gain more insights about the effectiveness of the two approaches.

Another area that needs to be investigated pertains to strategies to attract and retain volunteer vision guardians. Our experience is that, to sustain the project, we need to have a fixed number of paid vision guardians (typically one for a population unit of 5,000) in addition to volunteer vision guardians. A cluster of ten paid vision guardians can be monitored by a community supervisor, who will also engage with the volunteer vision guardians in the catchment area from time to time. This hybrid model, using a combination of paid and volunteer approaches, is a helpful way to achieve universal eye health in a given region.

**Figure:**
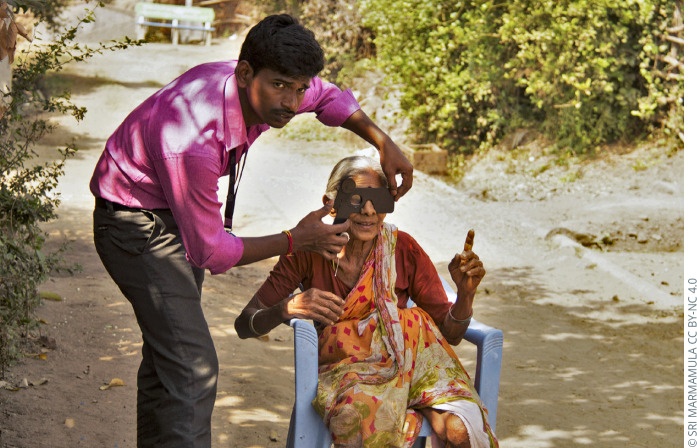
Visual acuity assessment. india

## References

[1] Bourne R Steinmetz JD Flaxman S Briant PS Taylor HR Resnikof S. Trends in prevalence of blindness and distance and near vision impairment over 30 years: an analysis for the Global Burden of Disease Study. Lancet Glob Health. 2021;9:e130-e143. 33275950 10.1016/S2214-109X(20)30425-3 PMC7820390

[2] Steinmetz JD Bourne RRA Briant PS Flaxman SR Taylor HRB Jonas JB. Causes of blindness and vision impairment in 2020 and trends over 30 years, and prevalence of avoidable blindness in relation to VISION 2020: the Right to Sight: an analysis for the Global Burden of Disease Study. Lancet Glob Health. 2021;9:e144-e160. 33275949 10.1016/S2214-109X(20)30489-7 PMC7820391

[3] World Health Organization. Universal eye health: a global action plan 2014-2019. Geneva: World Health Organization; 2013:28.

[4] Rao GN Khanna RC Athota SM Rajshekar V Rani PK. Integrated model of primary and secondary eye care for underserved rural areas: the LV Prasad Eye Institute experience. Indian J Ophthalmol. 2012;60:396-400. 22944748 10.4103/0301-4738.100533 PMC3491264

[5] Kuruganti S Mekala J Williams JD Krishnaiah S Rao GN Rani PK. Preventive eye health approach and elimination of avoidable blindness in remote rural areas – a vision health guardian approach. Rural Remote Health. 2012;12:1912. 22409251

[6] Marmamula S. The Basic Eye Screening Test (BEST) for primary level eye screening by grassroot level workers in India. Indian J Ophthalmol. 2020;68:408-9. 31957740 10.4103/ijo.IJO_1554_19 PMC7003602

[7] Rathi VM Williams JD Rajshekar V Khanna RC Das T. Tribal Odisha Eye Disease Study (TOES). Report # 10. Disability inclusive eye health survey in a tribal district (Rayagada) in Odisha, India. Indian J Ophthalmol. 2022;70:976-81. 35225555 10.4103/ijo.IJO_1887_21 PMC9114597

